# The *IKZF1* N159S mutation is associated with poor outcome and a distinct molecular profile in adult patients with AML

**DOI:** 10.1111/bjh.20027

**Published:** 2025-03-05

**Authors:** Sebastian Stasik, Jan‐Niklas Eckardt, Christoph Röllig, Claudia D. Baldus, Uwe Platzbecker, Hubert Serve, Carsten Müller‐Tidow, Kerstin Schäfer‐Eckart, Martin Kaufmann, Stefan W. Krause, Tim Sauer, Mathias Hänel, Andreas Neubauer, Gerhard Ehninger, Martin Bornhäuser, Johannes Schetelig, Jan M. Middeke, Christian Thiede, the Study Alliance Leukemia (SAL)

**Affiliations:** ^1^ Medizinische Klinik und Poliklinik I Universitätsklinikum Carl Gustav Carus Dresden Germany; ^2^ Klinik für Innere Medizin II, Hämatologie und Onkologie Universitätsklinikum Schleswig‐Holstein Kiel Germany; ^3^ Medizinische Klinik und Poliklinik I Universitätsklinikum Leipzig Leipzig Germany; ^4^ Medizinische Klinik II Universitätsklinikum Frankfurt Frankfurt am Main Germany; ^5^ Medizinische Klinik V Universitätsklinikum Heidelberg Heidelberg Germany; ^6^ Klinik für Innere Medizin V Klinikum Nürnberg Nord Nürnberg Germany; ^7^ Abteilung für Hämatologie, Onkologie und Palliativmedizin Robert‐Bosch‐Krankenhaus Stuttgart Germany; ^8^ Medizinische Klinik 5 Universitätsklinikum Erlangen Erlangen Germany; ^9^ Medizinische Klinik III Klinikum Chemnitz Chemnitz Germany; ^10^ Klinik für Hämatologie, Onkologie, Immunologie Philipps Universität Marburg Marburg Germany; ^11^ National Center for Tumor Diseases Dresden Germany; ^12^ DKMS Clinical Trials Unit Dresden Germany; ^13^ AgenDix GmbH Dresden Germany

**Keywords:** acute myeloid Leukaemia (AML), clinical outcome, *IKZF1*, molecular associations, N159S mutation

## Abstract

*IKZF1* mutations are recurrent alterations in acute myeloid leukaemia (AML), and hotspot point mutation, N159S, has recently been associated with unique gene expression and adverse risk. To better understand the molecular and clinical associations of *IKZF1* N159S‐mutated AML, we performed a pooled analysis of 4136 AML patients. *IKZF1* N159 mutations were found in 39 patients (0.94%) in a dominant clonal constellation, indicating early genetic events. N159S mutations were associated with aberrant karyotype, significantly higher rates of myelodysplasia‐related gene mutations, ELN2022 adverse risk and a particularly poor outcome, supporting the classification of *IKZF1* N159S‐mutated AML as a rare molecular subtype with adverse prognosis.

## INTRODUCTION

The Ikaros family zinc finger 1 (*IKZF1*) gene encodes the transcription factor IKAROS, a key regulator of haematopoiesis and lymphatic cell development, which mediates gene transcription via chromatin remodelling and epigenetic modification.^1^

Reduced expression of IKZF1 is associated with increased tumour immune evasion and progression in various malignancies, indicating its role as tumour suppressor and prognostic marker.^2^ Genomic alterations of *IKZF1* (e.g. deletions) were identified in haematological malignancies, like acute lymphoblastic leukaemia (ALL), with relevance for clinical outcome.^3^, ^4^, ^5^, ^6^, ^7^ In adult acute myeloid leukaemia (AML), genomic *IKZF1* alterations are reported in 2.6%–4.8% of patients, with a broader spectrum and distribution of variants.^4^, ^5^, ^6^ Recently, our group identified *IKZF1* mutations as an independent prognostic marker for adverse outcomes in a large multicentre cohort of AML patients. A mutational hotspot was the point mutation N159S (~40% of *IKZF1* mutations in AML) further designated *IKZF1* ^N159S^.^7^ The N159 residue is located in the conserved zinc finger 2 domain of *IKZF1*, relevant for DNA binding and transcriptional repression.^8^ Recent data showed that the *IKZF1* ^N159S^ mutation has a gain of function and inhibits wt‐IKZF1, indicating a leukaemogenic effect.^6^ To validate the clinical impact of *IKZF1* ^N159S^‐mutated AML, we screened 2530 additional adult AML patients for *IKZF1* N159 hotspot mutations. To better understand clinical and molecular associations, we performed a pooled analysis of 4136 AML patients, supporting the classification of *IKZF1* ^N159S^‐mutated AML as a rare but distinct AML subtype with adverse risk.

## METHODS

### Patients

To validate the clinical impact of *IKZF1* ^N159S^‐mutated AML, we retrospectively screened 2530 newly diagnosed and intensively treated adult AML patients from trials of the Study Alliance Leukaemia (Supporting Information) for *IKZF1* mutations at codon N159 (designated as the validation cohort). For detailed molecular characterization, we included 1606 AML patients^7^ with existing NGS panel data, including the *IKZF1* mutational status for a pooled analysis in 4136 AML patients.

### Molecular analysis

*IKZF1* ^N159S^ mutations were screened by Sanger sequencing on genomic DNA from bone marrow or peripheral blood taken at diagnosis. Validation of *IKZF1* ^N159S^ mutations and profiling of co‐mutations were performed using panel sequencing on a NextSeq NGS instrument (Illumina) (Supporting Information).

### Statistical analysis

As detailed in the supplement, variables between groups were compared using the chi‐squared, Mann–Whitney *U*‐test, or the Cochrane–Armitage test for trend for categorical variables with hierarchical order. *P*‐values <0.05 were considered statistically significant. Relapse‐free survival (RFS) and overall survival (OS) were evaluated using the Kaplan–Meier method and the log‐rank test. For multivariable analysis, Cox regression models were applied, using STATA 17 (StataCorp).

## RESULTS

### Validation of the clinical impact of *IKZF1* ^N159S^ AML

**FIGURE 1:**
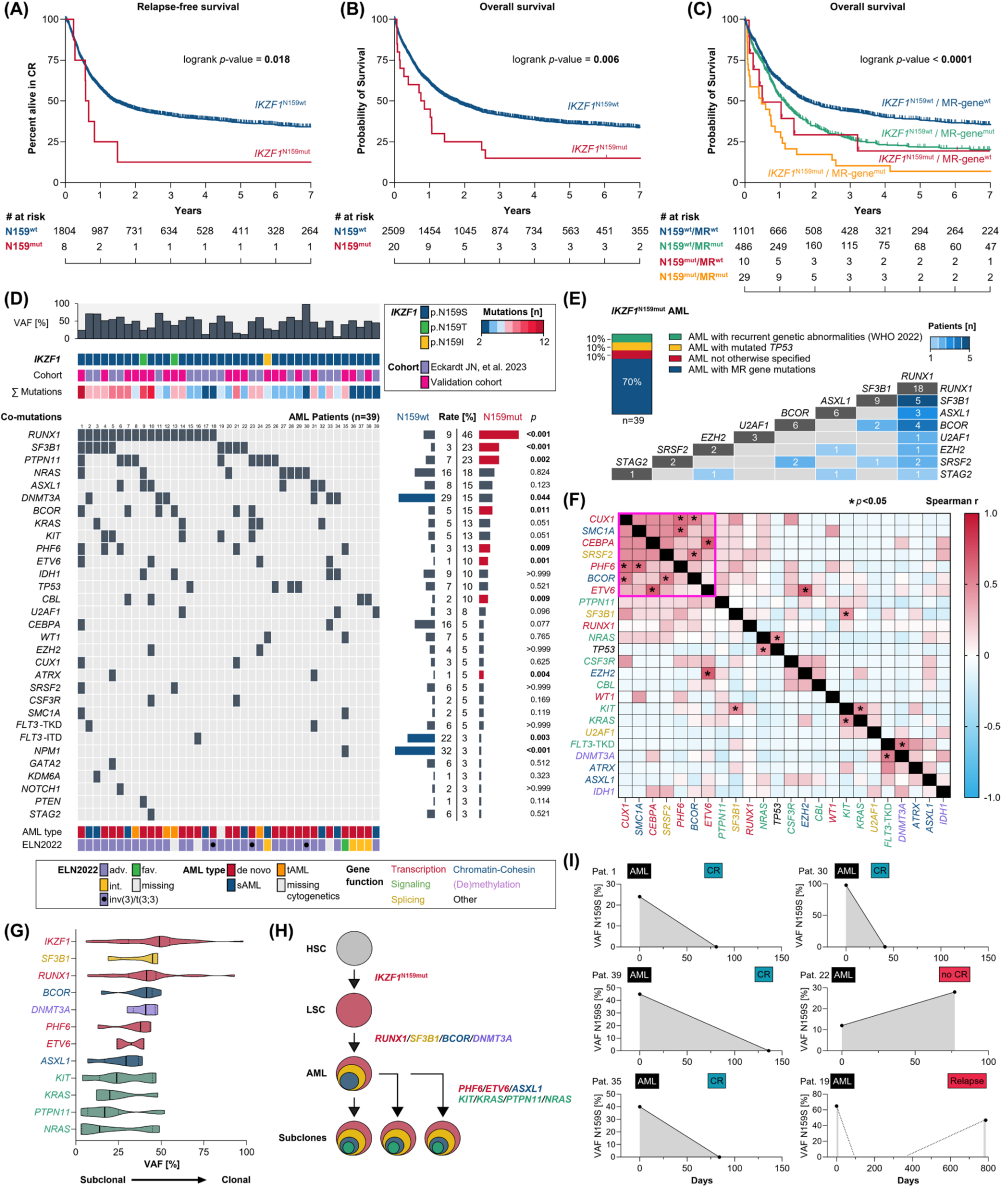
Molecular and clinical characteristics of *IKZF1* ^N159mut^ AML. Kaplan–Meier analysis showing the probability of (A) relapse‐free survival and (B) overall survival for *IKZF1* N159‐mutated (red) and wild‐type (blue) AML patients in the validation cohort of 2530 AML patients. (C) Kaplan–Meier analysis showing the probability of overall survival for different AML subgroups with or without *IKZF1* ^N159mut^/MR‐gene^mut^ (coded by colour) in AML patients with available NGS panel data (*n* = 1626/4136). (D) *IKZF1* variant allele frequencies (VAF), type of N159 mutation, overall number of co‐mutations (coded by colour) and rates of associated co‐mutations. (E) Grouping of *IKZF1* ^N159mut^ AMLs according to the WHO 2022 classification for AML as well as distribution and overlap across detected MR gene mutations. (F) Pairwise Spearman's rank correlation between gene mutations in *IKZF1* ^N159mut^ AMLs. The colour of each tile reflects the odds ratio for each pair. Blue colour indicates mutual exclusivity, while red colour indicates a positive correlation of co‐mutated pairs. Significant relationships are annotated by star (*; *p* < 0.05). (G) Distribution of VAFs of *IKZF1* ^N159mut^ and co‐mutated driver variants. Violin plots include a marker for median VAFs (solid line) and interquartile range (dashed line). (H) Schematic illustration of representative clonal hierarchies and sequential acquisition of genomic lesions, depicted from VAFs of *IKZF1* ^N159S^ and co‐mutations. (I) *IKZF1* ^N159S^ VAFs in matched samples taken at diagnosis and complete remission (or relapse).

Mutations at the *IKZF1* hotspot N159 residue (*IKZF1* ^N159mut^) were detected in 20 of 2530 AML patients (0.79%), with *IKZF1* ^N159S^ accounting for the vast majority of variants (in 17/20 cases) (Table 1; Table S1). Three patients showed the variant N159T (*n* = 2) or N159I (*n* = 1). *IKZF1* ^N159S^ status was associated with a lower frequency of ELN2017 favourable risk (5.9% vs. 25.8%; *p* = 0.032), a higher rate of aberrant karyotype (82.4% vs. 49.5%; *p* = 0.026) and lower bone marrow blast counts (40% vs. 60%; *p* = 0.027), compared to *IKZF1* ^N159wt^ AMLs (Table 1). No differences were observed for patients' age at diagnosis, AML type, the presence of a complex karyotype and other laboratory parameters. With respect to clinical outcome, *IKZF1* ^N159S^ status was associated with a lower rate of complete remission (CR; 47.1% vs. 71.9%; *p* = 0.030) and shorter (months; median) event‐free (EFS; 1.4 vs. 7; *p* = 0.004) and overall (OS; 8.4 vs. 21; *p* = 0.002) survival in univariable analyses (Table 1). Likewise, the *IKZF1* ^N159S^ status was an independent prognostic factor in multivariable analysis (odds/hazard ratio [95%‐CI]) for CR (0.26 [0.09–0.80]; *p* = 0.019), EFS (2.29 [1.40–3.76]; *p* = 0.001) and OS (1.98 [1.21–3.25]; *p* = 0.007) (Table S2). The rate of early death within 30 days of initial diagnosis (ED30) was significantly higher in *IKZF1* ^N159S^ AML patients (23.5% vs. 6.3%; *p* = 0.021), suggesting that increased induction deaths may contribute to CR failure in this subgroup. Similar to *IKZF1* ^N159S^, *IKZF1* ^N159mut^ status (N159T/I/S) was significantly associated with dismal outcome (including for RFS) in univariable (Figure 1A,B) and multivariable testing (Table 1; Table S3).

**TABLE 1:** Baseline patient characteristics with respect to *IKZF1* ^*N159*mut^ status.

Parameter	*IKZF1* ^*N159wt*^	*IKZF1* ^*N159mut*^	*p* ^a^	*IKZF1* ^*N159S*^	*p* ^a^
*n*/*N* (%)	2507^b^/2530 (99.1)	20/2530 (0.8)		17^c^/2530 (0.7)	
Age (years), median (IQR)	57 (46–67)	56 (36–63)	0.185	56 (36–61)	0.344
Sex, *n* (%)			0.659		0.812
Female	1236 (49.3)	11 (55.0)		9 (52.9)	
Male	1271 (50.7)	9 (45.0)		8 (47.1)	
Disease status, *n* (%)			0.586^e^		0.882^e^
de novo	1938 (77.3)	14 (70.0)		13 (76.4)	
sAML	352 (14.0)	3 (15.0)		2 (11.8)	
tAML	217 (8.7)	3 (15.0)		2 (11.8)	
Missing	0	0		0	
ELN2017 risk, *n* (%)			**0.037** ^f^		**0.029** ^f^
Favourable	647 (25.8)	1 (5.0)		1 (5.9)	
Intermediate	983 (39.2)	11 (55.0)		9 (52.9)	
Adverse	559 (22.3)	7 (35.0)		7 (41.2)	
Missing	318 (12.7)	1 (5.0)		0	
Complex karyotype, *n* (%)			0.197		0.165
No	1928 (76.9)	14 (70.0)		12 (70.6)	
Yes	349 (13.9)	5 (25.0)		5 (28.4)	
Missing	230 (9.2)	1 (5.0)		0	
Normal karyotype, *n* (%)			**0.037**		**0.026**
No	1240 (49.5)	15 (75.0)		14 (82.4)	
Yes	1048 (41.8)	4 (20.0)		3 (17.6)	
Missing	219 (8.7)	1 (5.0)		0	
allo HSCT in first CR, *n* (%)			1.000		0.754
No	2037 (81.3)	17 (85.0)		15 (88.2)	
Yes	470 (18.7)	3 (15.0)		2 (11.8)	
Missing	0	0		0	
Laboratory, median (IQR)					
WBC (10^9^/L)	9.4 (2.7–39.4)	7.9 (2.3–37.8)	0.616	11.2 (2.4–46.7)	0.182
HB (mmol/L)	5.6 (4.9–6.5)	5.8 (4.9–6.5)	0.745	5.3 (4.9–6.2)	0.723
PLT (10^9^/L)	50 (37–110)	54 (29–102)	0.457	64 (39–115)	0.195
PBB (%)	24 (5–60)	9 (1–41)	0.061	11 (2–42)	0.237
BMB (%)	60 (36–80)	39 (28–53)	**0.007**	40 (29–56)	**0.027**
Outcome					
Early death^d^, *n* (%)	159 (6.3)	4 (20.0)	**0.036**	4 (23.5)	**0.021**
CR rate, *n* (%)	1802 (71.9)	10 (50.0)	**0.037**	8 (47.1)	**0.030**
EFS, months median (95%‐CI)	7.0 (6.3–7.8)	1.4 (0.7–6.3)	**0.002**	1.4 (0.7–5.3)	**0.004**
RFS, months median (95%‐CI)	16.7 (15.1–19.9)	6.8 (2.8–10.0)	**0.018**	6.9 (2.8–17.8)	0.089
OS, months median (95%‐CI)	21.0 (18.4–23.0)	9.0 (1.7–17.1)	**0.006**	8.4 (1.0–17.1)	**0.002**

*Note*: Boldface indicates statistical significance (*p* < 0.05).

Abbreviations: 95%‐CI, 95% confidence interval; allo, allogeneic; AML, acute myeloid leukaemia; BMB, bone marrow blasts; CR, complete remission; EFS, event‐free survival; HB, haemoglobin; HSCT, haematopoietic stem cell transplantation; IQR, interquartile range; *n*/*N*, number; OS, overall survival; PBB, peripheral blood blasts; PLT, platelet count; RFS, relapse‐free survival; sAML, secondary AML; tAML, therapy‐associated AML; WBC, white blood cell count; wt, wild type.

^a^ Comparisons are made to *IKFZ1*‐N159 wild type.

^b^ Three patients had *IKZF1* alterations in exon 5 that were detected outside the N159 locus. These were G158S, Q146K and an indel at G190 resulting in a premature truncation.

^c^ Two patients had *IKZF1*‐N159T, and one patient had *IKZF1*‐N159I.

^d^ Early death was defined as death from any cause within 30 days after initial diagnosis.

^e^ Calculated with chi‐squared test.

^f^ Calculated with the Cochran–Armitage test for trend.

### Molecular associations of *IKZF1* ^N159mut^ AML

In a pooled analysis of 4136 AMLs, a total of 39 patients (0.94%) harboured mutations at *IKZF1* N159, with a median VAF of 49% (range: 6%–98%) (Figure 1D). *IKZF1* ^N159mut^ status was associated with higher rates of mutations in genes such as *RUNX1* (46% vs. 9%; *p* < 0.001), *SF3B1* (23% vs. 3%; *p* < 0.001) and *PTPN11* (23% vs. 7%; *p* = 0.002%). Vice versa, *IKZF1* ^N159wt^ status was associated with higher rates of *DNMT3A* (29% vs. 15%; *p* = 0.044), *FLT3*‐ITD (22% vs. 3%; *p* = 0.003) and *NPM1* (32% vs. 3%; *p* < 0.001) mutations (Figure 1D). Hierarchical classification according to cytogenetic and molecular abnormalities defined in the WHO 2022 classification of AML^9^ showed that *IKZF1* ^N159mut^ AML largely lacks subgroup‐defining genetic lesions, such as characteristic fusion genes or bZIP in‐frame *CEBPA* mutations (Figure 1E). Consequently, only four AML patients were classified under "AML with recurrent genetic abnormalities", including three patients with *MECOM*‐rearranged AML (inv(3)/t(3;3)), which was previously associated with *IKZF1* ^N159S^ mutations and adverse prognosis.^10^ Instead, 70% of *IKZF1* ^N159mut^ patients could be classified as "AML with myelodysplasia‐related gene mutations" (AML‐MR), mainly by a high rate of concomitant *RUNX1* mutations. Accordingly, AML patients with *IKZF1* ^N159mut^ status had a higher rate of ELN2022 adverse risk (86% vs. 36%; *p* < 0.001) compared to *IKZF1* ^N159wt^ AMLs. However, multivariable analysis adjusting for ELN2022 risk demonstrated that the *IKZF1* ^N159mut^ genotype represents an independent prognostic factor for poor outcome (Tables S4 and S5). Likewise, a subgroup analysis within AML‐MR patients confirmed a poor outcome of *IKZF1* ^N159mut^ patients (median OS: 5.68 vs. 13.37 months) even within this adverse risk category (Figure 1C). Interestingly, particularly poor survival rates were detected in those AML patients having both *IKZF1* ^N159mut^ and AML‐MR gene mutations. With respect to the molecular profile, the pairwise correlation of co‐mutated genes also revealed significant interactions across genes associated with transcriptional regulation (*CUX1*, *PHF6* and *ETV6*), chromatin‐cohesin modulation (*SMC1A* and *BCOR*) and splicing (*SRSF2*) (Figure 1F). *IKZF1* ^N159mut^ was generally detected at higher variant allele frequencies (VAFs) compared to associated co‐mutations (Figure 1G; Figure S1). Moreover, the analysis of material obtained at CR (or relapse) showed significant variations of N159S allelic burden (i.e. decrease below detection limits in CR), which demonstrates that *IKZF1* ^N159S^ mutations in these AMLs were somatically acquired during malignant progression (Figure 1I).

## DISCUSSION

Based on the largest cohort analysed for this alteration, we validated *IKZF1* ^N159S^ as a rare but recurrent genetic lesion in AML detected in the range of previous estimates (1.1%–1.89%).^6^, ^7^, ^11^ Besides AML, *IKZF1* ^N159S^ hotspot mutations were observed in patients with T‐cell acute lymphoblastic leukemia (T‐ALL)^12^ and in a combined immunodeficiency syndrome, where germline *IKZF1* ^N159S^ mutations exerted a dominant‐negative effect on IKAROS wild‐type function.^8^ For AML, we demonstrate that somatically acquired *IKZF1* ^N159S^ mutations are an independent prognostic marker for poor clinical outcome, associated with a specific co‐mutational profile, which is also different from AML patients with other *IKZF1* mutations.^6^, ^7^

For example, high rates of concomitant *RUNX1* and *SF3B1* alteration, but exclusivity for other common AML mutations (*CEBPA* bZIP in‐frame, *NPM1*, *FLT3*‐ITD), confirm recent findings for *IKZF1* ^N159S^‐mutated AML and are consistent with transcriptomic‐based evidence for a rare molecular AML subtype within the AML‐MR group.^6^, ^11^ More specifically, *IKZF1* ^N159S^ mutations were associated with the upregulation of oncogenic cell signalling pathways (i.e. *MYC*), abnormal genome binding patterns and a more aggressive phenotype in AML.^6^ In contrast, non‐N159S *IKZF1* mutations were previously associated with higher rates of *CEBPA* mutations^5^, ^7^ and a gene expression profile similar to biallelic *CEBPA* AMLs.^6^ Accordingly, our group recently observed a trend for inferior outcome in AML with *IKZF1* ^N159S^, compared to other *IKZF1* variants.^7^ Regarding the association with MR gene mutations, we showed that the adverse risk of AML‐MR^9^ is further increased by concomitant *IKZF1* ^N159S^ mutations. Although we observed a high frequency of MR gene mutations, we did not see an increased rate of secondary AML.^9^ Lower rates of bone marrow blasts and ELN favourable risk in *IKZF1* ^N159S^ AML likely reflect associations with chromatin/spliceosome alterations and the low *NPM1* mutation frequency.^13^ Furthermore, a significant overlap with genomic alterations of various transcription factors is indicative for distinct gene–gene interactions, which might synergistically contribute to leukaemogenesis in this AML subtype, with the *IKZF1* ^N159S^ mutation likely representing the earliest critical event (Figure 1H). As a limitation, Sanger sequencing in the validation cohort may have missed small *IKZF1* ^N159S^ subclones (<15% VAF). However, NGS data indicate that ~90% of *IKZF1* ^N159S^ have VAFs >15%,^7^ which are readily detectable by Sanger analysis. Interestingly, nine AML patients had *IKZF1* ^N159S^ VAFs >60%, suggesting that microdeletions at 7p12.2 may underlie loss of heterozygosity (LOH),^14^ as none displayed monosomy 7. Furthermore, *IKZF1* mutations, including *IKZF1* ^N159S^, are linked to HOXA/B, MEIS1 overexpression and MLL1/MENIN dysregulation.^6^, ^15^ Therefore, combined IKAROS degradation with immunomodulatory imide drugs and MENIN inhibition^15^ may offer an attractive therapeutic strategy for *IKZF1* ^N159S^ AML. Collectively, molecular data from this large AML cohort support *IKZF1* ^N159S^ mutations as a subgroup defining lesion with a dismal impact on the clinical course of AML, potentially modified by MR gene alterations.

## AUTHOR CONTRIBUTIONS

C.T. and S.S. were involved in conception of the work, acquisition/analysis of data and interpretation of data; J.‐N.E. and S.S. were involved in statistical analysis; S.S. were involved in bioinformatic analysis and drafted the manuscript; G.E. and M.B. were involved in administrative support; all authors involved in sample/data collection and have seen and approved the manuscript being submitted.

## FUNDING INFORMATION

This research did not receive any third‐party funding.

## CONFLICT OF INTEREST STATEMENT

C.T. is the CEO and co‐owner of AgenDix GmbH, a company performing molecular diagnostics. The remaining authors declare no conflict of interest.

## ETHICAL APPROVAL

All samples were collected with written informed consent and after approval by the local ethics committee of the Medical Faculty Carl Gustav Carus Dresden, Germany.

## CONSENT TO PARTICIPATE

The study protocols were designed and applied in accordance with the principles of the Declaration of Helsinki.

## CLINICAL TRIAL REGISTRATION

All patients were treated with intensive regimens in the following clinical trials: AML96 (NCT00180115), AML2003 (NCT00180102), AML60+ (NCT00180167) and SORAML (NCT00893373) or were enrolled in the German Study Alliance Leukaemia (SAL)'s AML registry (NCT03188874).

## PERMISSION TO REPRODUCE MATERIAL FROM OTHER SOURCES

I confirm that no material requiring permission to reproduce from other sources has been included in the paper.

## Supporting information


Data S1.

## Data Availability

Analysed datasets are available upon reasonable request from the corresponding author via email.
