# Supplementary material for: The IKZF1 N159S mutation is associated with poor outcome and a distinct molecular profile in adult patients with AML
Source: Br J Haematol. 2025 Mar 5;206(5):1373–9. doi: 10.1111/bjh.20027 (PMC12078884; doi:10.1111/bjh.20027)
Supplement: Supplementary file 1 — Data S1. [file BJH-206-1373-s001.zip › Table S2.docx]

| **Complete remission** | **OR [95%-CI]** | ***p*** |
| --- | --- | --- |
| *IKZF1*^N159S^ | 0.26 [0.09-0.80] | **0.019** |
| age | 0.94 [0.93-0.95] | **<0.001** |
| ELN2017 favorable risk | 1.69 [1.41-2.01] | **<0.001** |
| ELN2017 intermediate risk | 1.03 [0.88-1.21] | 0.689 |
| ELN2017 adverse risk | 0.50 [0.42-0.58] | **<0.001** |
| *de novo* AML | 1.68 [1.23-2.29] | **0.001** |
| sAML | 1.29 [0.89-1.87] | 0.173 |
| **Event-free survival** | **HR [95%-CI]** | ***p*** |
| *IKZF1*^N159S^ | 2.29 [1.40-3.76] | **0.001** |
| age | 1.02 [1.02-1.02] | **<0.001** |
| ELN2017 favorable risk | 0.63 [0.58-0.68] | **<0.001** |
| ELN2017 intermediate risk | 1.08 [1.00-1.16] | **0.046** |
| ELN2017 adverse risk | 1.64 [1.51-1.78] | **<0.001** |
| *de novo* AML | 0.97 [0.83-1.14] | 0.695 |
| sAML | 0.94 [0.78-1.14] | 0.545 |
| **Relapse-free survival** | **HR [95%-CI]** | ***p*** |
| *IKZF1*^N159S^ | 1.79 [0.85-3.78] | 0.125 |
| age | 1.02 [1.02-1.03] | **<0.001** |
| ELN2017 favorable risk | 0.69 [0.43-0.77] | **<0.001** |
| ELN2017 intermediate risk | 1.05 [0.96-1.16] | 0.300 |
| ELN2017 adverse risk | 1.54 [1.39-1.70] | **<0.001** |
| *de novo* AML | 0.99 [0.78-1.70] | 0.907 |
| sAML | 0.86 [0.65-1.13] | 0.276 |
| **Overall survival** | **HR [95%-CI]** | ***p*** |
| *IKZF1*^N159S^ | 1.98 [1.21-3.25] | **0.007** |
| age | 1.03 [1.03-1.04] | **<0.001** |
| ELN2017 favorable risk | 0.64 [0.58-0.70] | **<0.001** |
| ELN2017 intermediate risk | 1.02 [0.94-1.11] | 0.579 |
| ELN2017 adverse risk | 1.81 [1.66-1.97] | **<0.001** |
| *de novo* AML | 0.77 [0.66-0.91] | **0.003** |
| sAML | 0.76 [0.53-0.93] | **0.007** |

**Table S2** Summary of patient outcome with respect to *IKZF1* N159S mutation status in multivariable analyses. Square brackets show 95%-confidence intervals. Boldface indicates statistical significance (*p*<0.05). Abbreviations: hazard ratio (HR), odds ratio (OR), secondary AML (sAML).
