# Supplementary material for: The IKZF1 N159S mutation is associated with poor outcome and a distinct molecular profile in adult patients with AML
Source: Br J Haematol. 2025 Mar 5;206(5):1373–9. doi: 10.1111/bjh.20027 (PMC12078884; doi:10.1111/bjh.20027)
Supplement: Supplementary file 1 — Data S1. [file BJH-206-1373-s001.zip › Table S3.docx]

| **Complete remission** | **OR [95%-CI]** | ***p*** |
| --- | --- | --- |
| *IKZF1*^N159mut^ | 0.28 [0.10-0.80] | **0.017** |
| age | 0.94 [0.93-0.95] | **<0.001** |
| ELN2017 favorable risk | 1.68 [1.41-2.01] | **<0.001** |
| ELN2017 intermediate risk | 1.03 [0.88-1.21] | 0.680 |
| ELN2017 adverse risk | 0.49 [0.42-0.58] | **<0.001** |
| *de novo* AML | 1.68 [1.23-2.30] | **0.001** |
| sAML | 1.30 [0.90-1.88] | 0.159 |
| **Event-free survival** | **HR [95%-CI]** | ***p*** |
| *IKZF1*^N159mut^ | 2.39 [1.52-3.77] | **<0.001** |
| age | 1.02 [1.02-1.02] | **<0.001** |
| ELN2017 favorable risk | 0.63 [0.58-0.68] | **<0.001** |
| ELN2017 intermediate risk | 1.08 [1.00-1.16] | **0.045** |
| ELN2017 adverse risk | 1.64 [1.51-1.77] | **<0.001** |
| *de novo* AML | 0.96 [0.82-1.13] | 0.657 |
| sAML | 0.94 [0.78-1.13] | 0.518 |
| **Relapse-free survival** | **HR [95%-CI]** | ***p*** |
| *IKZF1*^N159mut^ | 2.16 [1.12-4.18] | **0.022** |
| age | 1.02 [1.02-1.03] | **<0.001** |
| ELN2017 favorable risk | 0.69 [0.63-0.77] | **<0.001** |
| ELN2017 intermediate risk | 1.05 [0.96-1.16] | 0.277 |
| ELN2017 adverse risk | 1.53 [1.38-1.70] | **<0.001** |
| *de novo* AML | 0.99 [0.79-1.24] | 0.919 |
| sAML | 0.86 [0.66-1.13] | 0.290 |
| **Overall survival** | **HR [95%-CI]** | ***p*** |
| *IKZF1*^N159mut^ | 1.88 [1.18-3.00] | **0.008** |
| age | 1.04 [1.03-1.04] | **<0.001** |
| ELN2017 favorable risk | 0.64 [0.58-0.70] | **<0.001** |
| ELN2017 intermediate risk | 1.02 [0.94-1.11] | 0.592 |
| ELN2017 adverse risk | 1.81 [1.66-1.97] | **<0.001** |
| *de novo* AML | 0.78 [0.66-0.92] | **0.003** |
| sAML | 0.76 [0.63-0.93] | **0.007** |

**Table S3** Summary of patient outcome with respect to *IKZF1* N159S/T/I mutation status in multivariable analyses. Square brackets show 95%-confidence intervals. Boldface indicates statistical significance (*p*<0.05). Abbreviations: hazard ratio (HR), odds ratio (OR), secondary AML (sAML).
