# Supplementary material for: The IKZF1 N159S mutation is associated with poor outcome and a distinct molecular profile in adult patients with AML
Source: Br J Haematol. 2025 Mar 5;206(5):1373–9. doi: 10.1111/bjh.20027 (PMC12078884; doi:10.1111/bjh.20027)
Supplement: Supplementary file 1 — Data S1. [file BJH-206-1373-s001.zip › Table S4.docx]

| **Complete remission** | **OR [95%-CI]** | ***p*** |
| --- | --- | --- |
| *IKZF1*^N159mut^ | 0.47 [0.22-0.98] | **0.045** |
| Age | 0.95 [0.94-0.96] | **<0.001** |
| ELN2022 favorable risk | 2.92 [1.81-4.71] | **<0.001** |
| ELN2022 intermediate risk | 1.47 [0.92-2.34] | 0.103 |
| ELN2022 adverse risk | 0.55 [0.36-0.85] | **0.007** |
| *de novo* AML | 1.86 [1.09-3.16] | **0.022** |
| sAML | 1.69 [0.93-3.08] | 0.084 |
| **Event-free survival** | **HR [95%-CI]** | ***p*** |
| *IKZF1*^N159mut^ | 1.68 [1.05-2.70] | **0.031** |
| Age | 1.02 [1.02-1.03] | **<0.001** |
| ELN2022 favorable risk | 0.53 [0.42-0.66] | **<0.001** |
| ELN2022 intermediate risk | 0.95 [0.76-1.19] | 0.674 |
| ELN2022 adverse risk | 1.56 [1.26-1.93] | **<0.001** |
| *de novo* AML | 0.89 [0.68-1.17] | 0.408 |
| sAML | 0.82 [0.61-1.11] | 0.198 |
| **Relapse-free survival** | **HR [95%-CI]** | ***p*** |
| *IKZF1*^N159mut^ | 1.64 [0.96-2.82] | 0.071 |
| Age | 1.02 [1.02-1.03] | **<0.001** |
| ELN2022 favorable risk | 0.58 [0.43-0.77] | **<0.001** |
| ELN2022 intermediate risk | 0.98 [0.73-1.33] | 0.914 |
| ELN2022 adverse risk | 1.29 [0.96-1.74] | 0.093 |
| *de novo* AML | 1.08 [0.71-1.64] | 0.720 |
| sAML | 0.97 [0.61-1.55] | 0.905 |
| **Overall survival** | **HR [95%-CI]** | ***p*** |
| *IKZF1*^N159mut^ | 1.67 [1.18-2.35] | **0.004** |
| Age | 1.03 [1.03-1.04] | **<0.001** |
| ELN2022 favorable risk | 0.56 [0.44-0.72] | **<0.001** |
| ELN2022 intermediate risk | 1.00 [0.78-1.27] | 0.969 |
| ELN2022 adverse risk | 1.50 [1.19-1.89] | **0.001** |
| *de novo* AML | 0.79 [0.60-1.04] | 0.098 |
| sAML | 0.77 [0.57-1.05] | 0.096 |

**Table S4** Summary of patient outcome with respect to *IKZF1* N159S/T/I mutation status in multivariable analyses. Square brackets show 95%-confidence intervals. Boldface indicates statistical significance (*p*<0.05). Abbreviations: hazard ratio (HR), odds ratio (OR), secondary AML (sAML).
