# Supplementary material for: The IKZF1 N159S mutation is associated with poor outcome and a distinct molecular profile in adult patients with AML
Source: Br J Haematol. 2025 Mar 5;206(5):1373–9. doi: 10.1111/bjh.20027 (PMC12078884; doi:10.1111/bjh.20027)
Supplement: Supplementary file 1 — Data S1. [file BJH-206-1373-s001.zip › BJH20027-sup-0001-Supplementary-Material.docx]

**Supplementary Apendix to:**

The *IKZF1* N159S mutation is associated with poor outcome and a distinct molecular profile in adult patients with AML

**Supplementary Methods**

*Patients*

We retrospectively analyzed a multicenter cohort of 4136 AML patients. Eligibility criteria were newly diagnosed AML according to WHO definitions [1], age ≥ 18 years, and available biomaterial at diagnosis. All patients were treated in the following clinical trials: AML96 [2], AML2003 [3], AML60+ [4], and SORAML [5] or were enrolled in the German Study Alliance Leukemia (SAL)’s AML registry (NCT03188874). All patients in our analysis received intensive induction regimens, and no additional treatments such as hypomethylating agents or targeted therapy were applied during induction. Detailed information on treatment regimens is given in the respective references. All mentioned studies were carried out under the auspices of the SAL, and approved by the Institutional Review Board of the Dresden University of Technology (Dresden, Saxony, Germany). All participants gave their written, informed consent, in accordance with to the Declaration of Helsinki.

*Sanger sequencing for the IKZF1*^N159S^ *hotspot mutation*

All mutational studies were performed on DNA from bone marrow aspirates or peripheral blood taken at diagnosis. DNA was extracted using the DNeasy blood and tissue kit (Qiagen, Hilden, Germany) and quantified with the NanoDrop spectrophotometer. PCR for *IKZF1*^N159S^ (at *IKZF1* exon 5; c.476 A>G; chr7:50450292; hg19) was performed using the primer sequences (5′-TTCTCGTAGCATCGTCCTCA-3′ (*IKZF1*_N159_Fw) and 5′-CAGGGGACCTACCGGAGT-3′ (*IKZF1*_N159_Re) amplifying a 250bp fragment (annealing 57°C). PCR was carried out using the Qiagen Multiplex PCR Kit in a volume of 50 μl, consisting of a Multiplex PCR Mix (1x), Q solution (1x), 0.2 μM of each *IKZF1* primer and 10 ng of template DNA on a GeneAmp PCR System 9700 (Applied Biosystems, Foster City, USA). PCR products were sequenced on an automated sequencer ABI3130xl (Life Technologies, Darmstadt, Germany).

*NGS panel sequencing for the detection of associated co-mutations*

Validation of IKZF1 N159S/T/I positive samples and profiling of co-mutations was done by targeted resequencing using the TruSight Myeloid assay (Illumina, Chesterford, UK) covering 54 genes recurrently mutated in AML: *BCOR*, *BCORL1*, *CDKN2A*, *CEBPA*, *CUX1*, *DNMT3A*, *ETV6*, *EZH2*, *IKZF1*, *KDM6A*, *PHF6*, *RAD21*, *RUNX1*, *STAG2*, *ZRSR2*, *ABL1*, *ASXL1*, *ATRX*, *BRAF*, *CALR*, *CBL*, *CBLB*, *CBLC*, *CDKN2A*, *CSF3R*, *FBXW7*, *FLT3*, *GATA1*, *GATA2*, *GNAS*, *HRAS*, *IDH1*, *IDH2*, *JAK2*, *JAK3*, *KIT*, *KRAS*, *MLL*, *MPL*, *MYD88*, *NOTCH1*, *NPM1*, *NRAS*, *PDGFRA*, *PTEN*, *PTPN11*, *SETBP1*, *SF3B1*, *SMC1A*, *SMC3*, *SRSF2*, *TET2*, *TP53*, *U2AF1* and *WT1*. For each reaction, 50 ng of genomic DNA was used. Library preparation was done as recommended by the manufacturer (TruSight Myeloid Sequencing Panel Reference Guide 15054779 v02, Illumina). Samples were sequenced paired-end (150 bp PE) on NextSeq- (Illumina) or (300 bp PE) MiSeq-NGS platforms. Sequence data alignment of demultiplexed FastQ files, variant calling and filtering was done using the Sequence Pilot software package (JSI medical systems GmbH, Ettenheim, Germany) with default settings and a 5% variant allele frequency (VAF) mutation calling cut-off. Human genome build HG19 was used as reference genome for mapping algorithms.

**References**

[1] Khoury JD, Solary E, Abla O, et al. The 5th edition of the World Health Organization Classification of Haematolymphoid Tumours: Myeloid and Histiocytic/Dendritic Neoplasms. Leukemia. 2022 Jul;36(7):1703-1719. doi: 10.1038/s41375-022-01613-1

[2] Röllig C, Thiede C, Gramatzki M, et al. A novel prognostic model in elderly patients with acute myeloid leukemia: results of 909 patients entered into the prospective AML96 trial. Blood. 2010 Aug 12;116(6):971-8. doi: 10.1182/blood-2010-01-267302.

[3] Schaich M, Parmentier S, Kramer M, et al. High-dose cytarabine consolidation with or without additional amsacrine and mitoxantrone in acute myeloid leukemia: results of the prospective randomized AML2003 trial. J Clin Oncol. 2013 Jun 10;31(17):2094-102. doi: 10.1200/JCO.2012.46.4743.

[4] Röllig C, Kramer M, Gabrecht M, et al. Intermediate-dose cytarabine plus mitoxantrone versus standard-dose cytarabine plus daunorubicin for acute myeloid leukemia in elderly patients. Ann Oncol. 2018 Apr 1;29(4):973-978. doi: 10.1093/annonc/mdy030.

[5] Röllig C, Serve H, Hüttmann A, et al. Addition of sorafenib versus placebo to standard therapy in patients aged 60 years or younger with newly diagnosed acute myeloid leukaemia (SORAML): a multicentre, phase 2, randomised controlled trial. Lancet Oncol. 2015 Dec;16(16):1691-9. doi: 10.1016/S1470-2045(15)00362-9.

**Statistical Analysis**

All tests were carried out as two-sided tests. Statistical significance was determined using a significance level α of 0.05. Variables between groups were compared using the Chi-squared or Mann-Whitney U test. For categorical variables with hierarchical order (ELN), the Cochrane-Armitage test for trend was used. The odds ratio (OR) for complete remission (CR) after intensive induction therapy was evaluated using logistic regression models. Time-to-event variables including event-free survival (EFS), relapse-free survival (RFS), and overall survival (OS), were analyzed using Cox proportional hazard models to obtain hazard ratios (HR) as well as the Kaplan-Meier method and the log-rank test. Competing hazards were evaluated using the method proposed by Fine and Gray (https://www.jstor.org/stable/2670170) and cumulative incidence of failure was subsequently plotted. For all OR and HR, 95%-confidence intervals (95%-CI) are reported. All analyses were performed and visualizations were created in STATA BE 18.0 (Stata Corp, College Station, TX, USA).

**Supplementary Data**

| **Patient** | **HGVSc** | **HGVSp** | **VAF** | **AML type** | **ELN2017 risk** | **Study cohort** |
| --- | --- | --- | --- | --- | --- | --- |
| 1 | c.476A>G | p.Asn159Ser | 24 | *de novo* | adverse | Validation cohort (present) |
| 2 | c.476A>G | p.Asn159Ser | 71 | sAML | adverse | Eckardt JN, et al. 2023 |
| 3 | c.476A>G | p.Asn159Ser | 70 | sAML | adverse | Eckardt JN, et al. 2023 |
| 4 | c.476A>G | p.Asn159Ser | 51 | *de novo* | intermediate | Validation cohort (present) |
| 5 | c.476A>G | p.Asn159Ser | 55 | *de novo* | adverse | Eckardt JN, et al. 2023 |
| 6 | c.476A>G | p.Asn159Ser | 62 | *de novo* | adverse | Eckardt JN, et al. 2023 |
| 7 | c.476A>G | p.Asn159Ser | 41 | sAML | intermediate | Validation cohort (present) |
| 8 | c.476A>G | p.Asn159Ser | 50 | tAML | adverse | Validation cohort (present) |
| 9 | c.476A>C | p.Asn159Thr | 24 | *de novo* | intermediate | Validation cohort (present) |
| 10 | c.476A>G | p.Asn159Ser | 31 | *de novo* | intermediate | Validation cohort (present) |
| 11 | c.476A>G | p.Asn159Ser | 73 | *de novo* | adverse | Eckardt JN, et al. 2023 |
| 12 | c.476A>G | p.Asn159Ser | 50 | tAML | intermediate | Validation cohort (present) |
| 13 | c.476A>C | p.Asn159Thr | 38 | tAML | missing | Validation cohort (present) |
| 14 | c.476A>G | p.Asn159Ser | 52 | *de novo* | adverse | Validation cohort (present) |
| 15 | c.476A>G | p.Asn159Ser | 62 | *de novo* | adverse | Eckardt JN, et al. 2023 |
| 16 | c.476A>G | p.Asn159Ser | 54 | *de novo* | missing | Eckardt JN, et al. 2023 |
| 17 | c.476A>G | p.Asn159Ser | 12 | sAML | adverse | Eckardt JN, et al. 2023 |
| 18 | c.476A>G | p.Asn159Ser | 44 | *de novo* | adverse | Eckardt JN, et al. 2023 |
| 19 | c.476A>G | p.Asn159Ser | 65 | missing | intermediate | Eckardt JN, et al. 2023 |
| 20 | c.476A>G | p.Asn159Ser | 48 | *de novo* | adverse | Validation cohort (present) |
| 21 | c.476A>G | p.Asn159Ser | 16 | *de novo* | intermediate | Validation cohort (present) |
| 22 | c.476A>G | p.Asn159Ser | 12 | sAML | adverse | Validation cohort (present) |
| 23 | c.476A>G | p.Asn159Ser | 49 | *de novo* | adverse | Eckardt JN, et al. 2023 |
| 24 | c.476A>G | p.Asn159Ser | 60 | tAML | intermediate | Eckardt JN, et al. 2023 |
| 25 | c.476A>T | p.Asn159Ile | 28 | sAML | intermediate | Validation cohort (present) |
| 26 | c.476A>G | p.Asn159Ser | 24 | *de novo* | adverse | Validation cohort (present) |
| 27 | c.476A>G | p.Asn159Ser | 49 | *de novo* | intermediate | Validation cohort (present) |
| 28 | c.476A>G | p.Asn159Ser | 61 | *de novo* | adverse | Eckardt JN, et al. 2023 |
| 29 | c.476A>G | p.Asn159Ser | 52 | *de novo* | adverse | Validation cohort (present) |
| 30 | c.476A>G | p.Asn159Ser | 98 | *de novo* | adverse | Eckardt JN, et al. 2023 |
| 31 | c.476A>G | p.Asn159Ser | 47 | *de novo* | intermediate | Validation cohort (present) |
| 32 | c.476A>G | p.Asn159Ser | 31 | sAML | adverse | Eckardt JN, et al. 2023 |
| 33 | c.476A>G | p.Asn159Ser | 50 | *de novo* | missing | Eckardt JN, et al. 2023 |
| 34 | c.476A>G | p.Asn159Ser | 6 | sAML | intermediate | Eckardt JN, et al. 2023 |
| 35 | c.476A>G | p.Asn159Ser | 40 | *de novo* | favourable | Validation cohort (present) |
| 36 | c.476A>G | p.Asn159Ser | 51 | *de novo* | intermediate | Validation cohort (present) |
| 37 | c.476A>G | p.Asn159Ser | 33 | *de novo* | adverse | Eckardt JN, et al. 2023 |
| 38 | c.476A>G | p.Asn159Ser | 50 | *de novo* | intermediate | Validation cohort (present) |
| 39 | c.476A>G | p.Asn159Ser | 45 | sAML | adverse | Eckardt JN, et al. 2023 |

**Table S1** *IKZF1*^N159mut^ AML patients; Abbreviations: HGVSc (HGVS coding sequence name), HGVSp (HGVS protein sequence name), VAF (variant allele frequency), ELN (European LeukemiaNet).

| **Complete remission** | **OR [95%-CI]** | ***p*** |
| --- | --- | --- |
| *IKZF1*^N159S^ | 0.26 [0.09-0.80] | **0.019** |
| age | 0.94 [0.93-0.95] | **<0.001** |
| ELN2017 favorable risk | 1.69 [1.41-2.01] | **<0.001** |
| ELN2017 intermediate risk | 1.03 [0.88-1.21] | 0.689 |
| ELN2017 adverse risk | 0.50 [0.42-0.58] | **<0.001** |
| *de novo* AML | 1.68 [1.23-2.29] | **0.001** |
| sAML | 1.29 [0.89-1.87] | 0.173 |
| **Event-free survival** | **HR [95%-CI]** | ***p*** |
| *IKZF1*^N159S^ | 2.29 [1.40-3.76] | **0.001** |
| age | 1.02 [1.02-1.02] | **<0.001** |
| ELN2017 favorable risk | 0.63 [0.58-0.68] | **<0.001** |
| ELN2017 intermediate risk | 1.08 [1.00-1.16] | **0.046** |
| ELN2017 adverse risk | 1.64 [1.51-1.78] | **<0.001** |
| *de novo* AML | 0.97 [0.83-1.14] | 0.695 |
| sAML | 0.94 [0.78-1.14] | 0.545 |
| **Relapse-free survival** | **HR [95%-CI]** | ***p*** |
| *IKZF1*^N159S^ | 1.79 [0.85-3.78] | 0.125 |
| age | 1.02 [1.02-1.03] | **<0.001** |
| ELN2017 favorable risk | 0.69 [0.43-0.77] | **<0.001** |
| ELN2017 intermediate risk | 1.05 [0.96-1.16] | 0.300 |
| ELN2017 adverse risk | 1.54 [1.39-1.70] | **<0.001** |
| *de novo* AML | 0.99 [0.78-1.70] | 0.907 |
| sAML | 0.86 [0.65-1.13] | 0.276 |
| **Overall survival** | **HR [95%-CI]** | ***p*** |
| *IKZF1*^N159S^ | 1.98 [1.21-3.25] | **0.007** |
| age | 1.03 [1.03-1.04] | **<0.001** |
| ELN2017 favorable risk | 0.64 [0.58-0.70] | **<0.001** |
| ELN2017 intermediate risk | 1.02 [0.94-1.11] | 0.579 |
| ELN2017 adverse risk | 1.81 [1.66-1.97] | **<0.001** |
| *de novo* AML | 0.77 [0.66-0.91] | **0.003** |
| sAML | 0.76 [0.53-0.93] | **0.007** |

**Table S2** Summary of patient outcome with respect to *IKZF1* N159S mutation status in multivariable analyses. Square brackets show 95%-confidence intervals. Boldface indicates statistical significance (*p*<0.05). Abbreviations: hazard ratio (HR), odds ratio (OR), secondary AML (sAML).

| **Complete remission** | **OR [95%-CI]** | ***p*** |
| --- | --- | --- |
| *IKZF1*^N159mut^ | 0.28 [0.10-0.80] | **0.017** |
| age | 0.94 [0.93-0.95] | **<0.001** |
| ELN2017 favorable risk | 1.68 [1.41-2.01] | **<0.001** |
| ELN2017 intermediate risk | 1.03 [0.88-1.21] | 0.680 |
| ELN2017 adverse risk | 0.49 [0.42-0.58] | **<0.001** |
| *de novo* AML | 1.68 [1.23-2.30] | **0.001** |
| sAML | 1.30 [0.90-1.88] | 0.159 |
| **Event-free survival** | **HR [95%-CI]** | ***p*** |
| *IKZF1*^N159mut^ | 2.39 [1.52-3.77] | **<0.001** |
| age | 1.02 [1.02-1.02] | **<0.001** |
| ELN2017 favorable risk | 0.63 [0.58-0.68] | **<0.001** |
| ELN2017 intermediate risk | 1.08 [1.00-1.16] | **0.045** |
| ELN2017 adverse risk | 1.64 [1.51-1.77] | **<0.001** |
| *de novo* AML | 0.96 [0.82-1.13] | 0.657 |
| sAML | 0.94 [0.78-1.13] | 0.518 |
| **Relapse-free survival** | **HR [95%-CI]** | ***p*** |
| *IKZF1*^N159mut^ | 2.16 [1.12-4.18] | **0.022** |
| age | 1.02 [1.02-1.03] | **<0.001** |
| ELN2017 favorable risk | 0.69 [0.63-0.77] | **<0.001** |
| ELN2017 intermediate risk | 1.05 [0.96-1.16] | 0.277 |
| ELN2017 adverse risk | 1.53 [1.38-1.70] | **<0.001** |
| *de novo* AML | 0.99 [0.79-1.24] | 0.919 |
| sAML | 0.86 [0.66-1.13] | 0.290 |
| **Overall survival** | **HR [95%-CI]** | ***p*** |
| *IKZF1*^N159mut^ | 1.88 [1.18-3.00] | **0.008** |
| age | 1.04 [1.03-1.04] | **<0.001** |
| ELN2017 favorable risk | 0.64 [0.58-0.70] | **<0.001** |
| ELN2017 intermediate risk | 1.02 [0.94-1.11] | 0.592 |
| ELN2017 adverse risk | 1.81 [1.66-1.97] | **<0.001** |
| *de novo* AML | 0.78 [0.66-0.92] | **0.003** |
| sAML | 0.76 [0.63-0.93] | **0.007** |

**Table S3** Summary of patient outcome with respect to *IKZF1* N159S/T/I mutation status in multivariable analyses. Square brackets show 95%-confidence intervals. Boldface indicates statistical significance (*p*<0.05). Abbreviations: hazard ratio (HR), odds ratio (OR), secondary AML (sAML).

| **Complete remission** | **OR [95%-CI]** | ***p*** |
| --- | --- | --- |
| *IKZF1*^N159mut^ | 0.47 [0.22-0.98] | **0.045** |
| Age | 0.95 [0.94-0.96] | **<0.001** |
| ELN2022 favorable risk | 2.92 [1.81-4.71] | **<0.001** |
| ELN2022 intermediate risk | 1.47 [0.92-2.34] | 0.103 |
| ELN2022 adverse risk | 0.55 [0.36-0.85] | **0.007** |
| *de novo* AML | 1.86 [1.09-3.16] | **0.022** |
| sAML | 1.69 [0.93-3.08] | 0.084 |
| **Event-free survival** | **HR [95%-CI]** | ***p*** |
| *IKZF1*^N159mut^ | 1.68 [1.05-2.70] | **0.031** |
| Age | 1.02 [1.02-1.03] | **<0.001** |
| ELN2022 favorable risk | 0.53 [0.42-0.66] | **<0.001** |
| ELN2022 intermediate risk | 0.95 [0.76-1.19] | 0.674 |
| ELN2022 adverse risk | 1.56 [1.26-1.93] | **<0.001** |
| *de novo* AML | 0.89 [0.68-1.17] | 0.408 |
| sAML | 0.82 [0.61-1.11] | 0.198 |
| **Relapse-free survival** | **HR [95%-CI]** | ***p*** |
| *IKZF1*^N159mut^ | 1.64 [0.96-2.82] | 0.071 |
| Age | 1.02 [1.02-1.03] | **<0.001** |
| ELN2022 favorable risk | 0.58 [0.43-0.77] | **<0.001** |
| ELN2022 intermediate risk | 0.98 [0.73-1.33] | 0.914 |
| ELN2022 adverse risk | 1.29 [0.96-1.74] | 0.093 |
| *de novo* AML | 1.08 [0.71-1.64] | 0.720 |
| sAML | 0.97 [0.61-1.55] | 0.905 |
| **Overall survival** | **HR [95%-CI]** | ***p*** |
| *IKZF1*^N159mut^ | 1.67 [1.18-2.35] | **0.004** |
| Age | 1.03 [1.03-1.04] | **<0.001** |
| ELN2022 favorable risk | 0.56 [0.44-0.72] | **<0.001** |
| ELN2022 intermediate risk | 1.00 [0.78-1.27] | 0.969 |
| ELN2022 adverse risk | 1.50 [1.19-1.89] | **0.001** |
| *de novo* AML | 0.79 [0.60-1.04] | 0.098 |
| sAML | 0.77 [0.57-1.05] | 0.096 |

**Table S4** Summary of patient outcome with respect to *IKZF1* N159S/T/I mutation status in multivariable analyses. Square brackets show 95%-confidence intervals. Boldface indicates statistical significance (*p*<0.05). Abbreviations: hazard ratio (HR), odds ratio (OR), secondary AML (sAML).

| **Complete remission** | **OR [95%-CI]** | ***p*** |
| --- | --- | --- |
| *IKZF1*^N159S^ | 0.45 [0.21-0.95] | **0.036** |
| Age | 0.95 [0.94-0.96] | **<0.001** |
| ELN2022 favorable risk | 2.92 [1.81-4.71] | **<0.001** |
| ELN2022 intermediate risk | 1.47 [0.92-2.33] | 0.104 |
| ELN2022 adverse risk | 0.55 [0.36-0.85] | **0.007** |
| *de novo* AML | 1.86 [1.09-3.16] | **0.022** |
| sAML | 1.69 [0.93-3.08] | 0.085 |
| **Event-free survival** | **HR [95%-CI]** | ***p*** |
| *IKZF1*^N159S^ | 1.68 [1.05-2.70] | **0.031** |
| Age | 1.02 [1.02-1.03] | **<0.001** |
| ELN2022 favorable risk | 0.53 [0.42-0.66] | **<0.001** |
| ELN2022 intermediate risk | 0.95 [0.76-1.19] | 0.674 |
| ELN2022 adverse risk | 1.56 [1.26-1.93] | **<0.001** |
| *de novo* AML | 0.89 [0.68-1.17] | 0.408 |
| sAML | 0.82 [0.61-1.11] | 0.198 |
| **Relapse-free survival** | **HR [95%-CI]** | ***p*** |
| *IKZF1*^N159S^ | 1.56 [0.89-2.73] | 0.119 |
| Age | 1.02 [1.02-1.03] | **<0.001** |
| ELN2022 favorable risk | 0.58 [0.43-0.77] | **<0.001** |
| ELN2022 intermediate risk | 0.98 [0.73-1.33] | 0.915 |
| ELN2022 adverse risk | 1.30 [0.96-1.74] | 0.089 |
| *de novo* AML | 1.08 [0.71-1.64] | 0.726 |
| sAML | 0.97 [0.61-1.55] | 0.907 |
| **Overall survival** | **HR [95%-CI]** | ***p*** |
| *IKZF1*^N159S^ | 1.65 [1.16-2.33] | **0.005** |
| Age | 1.03 [1.03-1.04] | **<0.001** |
| ELN2022 favorable risk | 0.56 [0.44-0.72] | **<0.001** |
| ELN2022 intermediate risk | 1.00 [0.78-1.27] | 0.968 |
| ELN2022 adverse risk | 1.50 [1.19-1.89] | **<0.001** |
| *de novo* AML | 0.79 [0.60-1.04] | 0.098 |
| sAML | 0.77 [0.57-1.05] | 0.097 |

**Table S5** Summary of patient outcome with respect to *IKZF1* N159S mutation status in multivariable analyses. Square brackets show 95%-confidence intervals. Boldface indicates statistical significance (*p*<0.05). Abbreviations: hazard ratio (HR), odds ratio (OR), secondary AML (sAML).


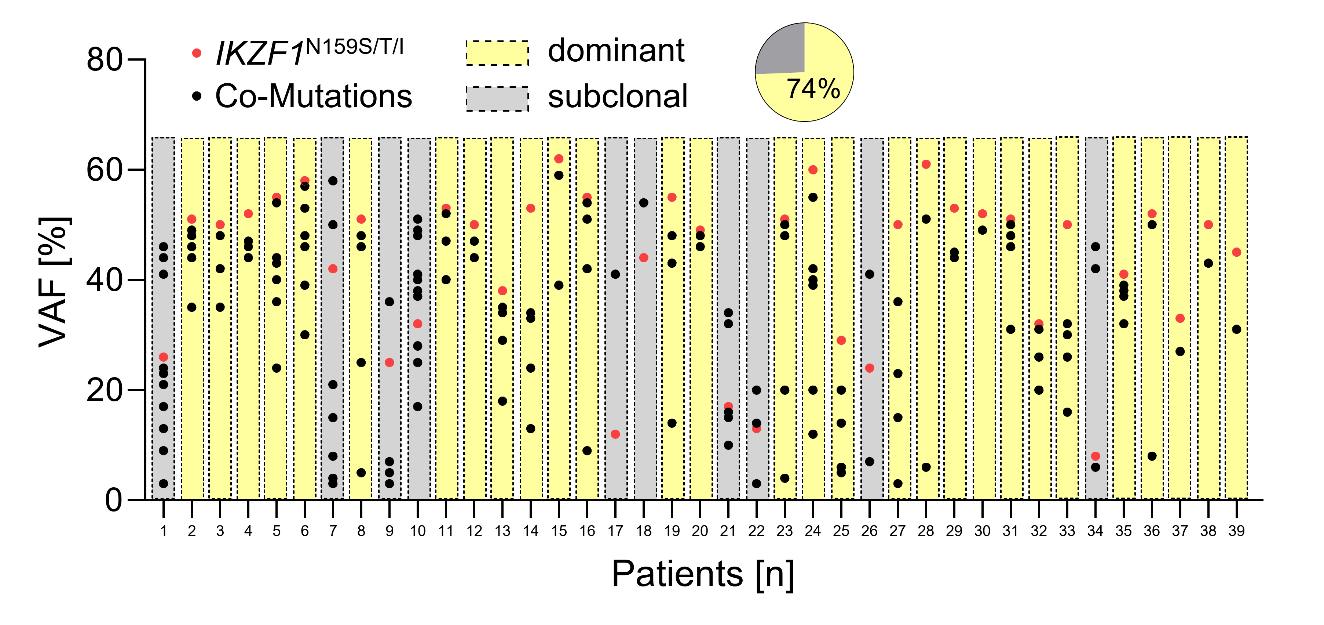


**Figure S1** Variant allele frequencies (VAFs) of *IKZF1*^N159S/T/I^ mutations (red) and co-mutated driver variants (black) detected in genomic DNA from bone marrow aspirates or peripheral blood taken at diagnosis in individual AML patients. Yellow bars represent AML cases with a dominant *IKZF1*^N159S/T/I^ mutation, while grey bars indicate cases with a subclonal configuration of *IKZF1*^N159S/T/I^ mutations.
