# Supplementary material for: The IKZF1 N159S mutation is associated with poor outcome and a distinct molecular profile in adult patients with AML
Source: Br J Haematol. 2025 Mar 5;206(5):1373–9. doi: 10.1111/bjh.20027 (PMC12078884; doi:10.1111/bjh.20027)
Supplement: Supplementary file 1 — Data S1. [file BJH-206-1373-s001.zip › Table S1.docx]

| **Patient** | **HGVSc** | **HGVSp** | **VAF** | **AML type** | **ELN2017 risk** | **Study cohort** |
| --- | --- | --- | --- | --- | --- | --- |
| 1 | c.476A>G | p.Asn159Ser | 24 | *de novo* | adverse | Validation cohort (present) |
| 2 | c.476A>G | p.Asn159Ser | 71 | sAML | adverse | Eckardt JN, et al. 2023 |
| 3 | c.476A>G | p.Asn159Ser | 70 | sAML | adverse | Eckardt JN, et al. 2023 |
| 4 | c.476A>G | p.Asn159Ser | 51 | *de novo* | intermediate | Validation cohort (present) |
| 5 | c.476A>G | p.Asn159Ser | 55 | *de novo* | adverse | Eckardt JN, et al. 2023 |
| 6 | c.476A>G | p.Asn159Ser | 62 | *de novo* | adverse | Eckardt JN, et al. 2023 |
| 7 | c.476A>G | p.Asn159Ser | 41 | sAML | intermediate | Validation cohort (present) |
| 8 | c.476A>G | p.Asn159Ser | 50 | tAML | adverse | Validation cohort (present) |
| 9 | c.476A>C | p.Asn159Thr | 24 | *de novo* | intermediate | Validation cohort (present) |
| 10 | c.476A>G | p.Asn159Ser | 31 | *de novo* | intermediate | Validation cohort (present) |
| 11 | c.476A>G | p.Asn159Ser | 73 | *de novo* | adverse | Eckardt JN, et al. 2023 |
| 12 | c.476A>G | p.Asn159Ser | 50 | tAML | intermediate | Validation cohort (present) |
| 13 | c.476A>C | p.Asn159Thr | 38 | tAML | missing | Validation cohort (present) |
| 14 | c.476A>G | p.Asn159Ser | 52 | *de novo* | adverse | Validation cohort (present) |
| 15 | c.476A>G | p.Asn159Ser | 62 | *de novo* | adverse | Eckardt JN, et al. 2023 |
| 16 | c.476A>G | p.Asn159Ser | 54 | *de novo* | missing | Eckardt JN, et al. 2023 |
| 17 | c.476A>G | p.Asn159Ser | 12 | sAML | adverse | Eckardt JN, et al. 2023 |
| 18 | c.476A>G | p.Asn159Ser | 44 | *de novo* | adverse | Eckardt JN, et al. 2023 |
| 19 | c.476A>G | p.Asn159Ser | 65 | missing | intermediate | Eckardt JN, et al. 2023 |
| 20 | c.476A>G | p.Asn159Ser | 48 | *de novo* | adverse | Validation cohort (present) |
| 21 | c.476A>G | p.Asn159Ser | 16 | *de novo* | intermediate | Validation cohort (present) |
| 22 | c.476A>G | p.Asn159Ser | 12 | sAML | adverse | Validation cohort (present) |
| 23 | c.476A>G | p.Asn159Ser | 49 | *de novo* | adverse | Eckardt JN, et al. 2023 |
| 24 | c.476A>G | p.Asn159Ser | 60 | tAML | intermediate | Eckardt JN, et al. 2023 |
| 25 | c.476A>T | p.Asn159Ile | 28 | sAML | intermediate | Validation cohort (present) |
| 26 | c.476A>G | p.Asn159Ser | 24 | *de novo* | adverse | Validation cohort (present) |
| 27 | c.476A>G | p.Asn159Ser | 49 | *de novo* | intermediate | Validation cohort (present) |
| 28 | c.476A>G | p.Asn159Ser | 61 | *de novo* | adverse | Eckardt JN, et al. 2023 |
| 29 | c.476A>G | p.Asn159Ser | 52 | *de novo* | adverse | Validation cohort (present) |
| 30 | c.476A>G | p.Asn159Ser | 98 | *de novo* | adverse | Eckardt JN, et al. 2023 |
| 31 | c.476A>G | p.Asn159Ser | 47 | *de novo* | intermediate | Validation cohort (present) |
| 32 | c.476A>G | p.Asn159Ser | 31 | sAML | adverse | Eckardt JN, et al. 2023 |
| 33 | c.476A>G | p.Asn159Ser | 50 | *de novo* | missing | Eckardt JN, et al. 2023 |
| 34 | c.476A>G | p.Asn159Ser | 6 | sAML | intermediate | Eckardt JN, et al. 2023 |
| 35 | c.476A>G | p.Asn159Ser | 40 | *de novo* | favourable | Validation cohort (present) |
| 36 | c.476A>G | p.Asn159Ser | 51 | *de novo* | intermediate | Validation cohort (present) |
| 37 | c.476A>G | p.Asn159Ser | 33 | *de novo* | adverse | Eckardt JN, et al. 2023 |
| 38 | c.476A>G | p.Asn159Ser | 50 | *de novo* | intermediate | Validation cohort (present) |
| 39 | c.476A>G | p.Asn159Ser | 45 | sAML | adverse | Eckardt JN, et al. 2023 |

**Table S1** *IKZF1*^N159mut^ AML patients; Abbreviations: HGVSc (HGVS coding sequence name), HGVSp (HGVS protein sequence name), VAF (variant allele frequency), ELN (European LeukemiaNet).
